# Diagnostic performance and agreement of auditors for evaluation of computer-aided optical polyp diagnosis: Prospective study

**DOI:** 10.1055/a-2631-8030

**Published:** 2025-07-01

**Authors:** Felix Huang, Thea Iulia Dimbu, Douglas K. Rex, Heiko Pohl, Cesare Hassan, Roupen Djinbachian, Victoire Michal, Dong Hyun Kim, Bilal Amani, Nahlah Haddouch, Sofie Fournier, Daniel von Renteln

**Affiliations:** 1 Research Center (CRCHUM) and Gastroenterology Division, Centre Hospitalier de l'Université de Montréal (CHUM), Montreal, Canada; 2 12368 Faculty of Medicine, Université de Montréal Faculté de Médecine, Montreal, Canada; 3 12250 Division of Gastroenterology/Hepatology, Indiana University School of Medicine, Indianapolis, United States; 4 Section of Gastroenterology, VA Medical Center, Vermont, United States; 5 437807 Department of Biomedical Sciences, Humanitas University, Milan, Italy; 6 Endoscopy Unit, IRCCS Humanitas Clinical and Research Center, Milan, Italy; 7 5620 Medicine, McGill University, Montreal, Canada

**Keywords:** Endoscopy Lower GI Tract, Polyps / adenomas / ..., GI Pathology, Quality and logistical aspects, Quality management

## Abstract

**Background and study aims:**

Guidelines recommend independent auditing of diagnostic performance for clinical implementation of computer-aided optical polyp diagnosis (CADx). This study evaluated diagnostic performance and interobserver agreement of auditors and offered guidance on conducting CADx audits.

**Methods:**

Images and videos of all ≤ 5-mm polyps from a large, prospective study with systematic activation of CADx were audited by three expert endoscopists. Experts performed independent, blinded diagnostic review including documentation of confidence level. The primary outcome was sensitivity of audit by three experts for high-confidence adenomas compared with pathology. Secondary outcomes included number of reviewers for optimal CADx auditing and interobserver agreement.

**Results:**

Four hundred eighty-seven diminutive polyps were audited (510 patients). Sensitivity was 99.4% (95% confidence interval [CI] 96.0–100) using three experts (Strategy A); 88.7% (95% CI 84.1–92.1) using two experts and one referee (Strategy B); 99% (95% CI 96–99.8), 98.8% (95% CI 95.4–99.8), and 99.4% (95% CI 96.3–100) using two-expert combinations (Strategy C); and 98.2% (95% CI 95.1–99.4), 97.3% (95% CI 94.0–98.9), and 88.9% (95% CI 83.6–92.7) for each expert individually (Strategy D). Among 266 pathology-based adenomas, Strategy A evaluated 160 polyps versus 196, 172, and 170 in Strategy C; and 220, 223, and 207 in Strategy D. Strategy B evaluated all 266 adenomas. Overall interobserver agreement was moderate (kappa 0.52), but very high for high-confidence adenomas (kappa 0.89).

**Conclusions:**

Expert audit for evaluating CADx resulted in high sensitivity and interobserver agreement for high-confidence adenomas. Audit by two experts, with a third expert for arbitration, permitted audit of all polyps and effective assessment of CADx within clinical studies.

## Introduction

Histopathology is currently considered the gold standard for diagnostic evaluation of diminutive (≤ 5 mm) colorectal polyps. Clinical implementation of real-time optical polyp diagnosis using computer-aided diagnosis systems (CADx) would allow histopathology to be replaced, reducing resource utilization and healthcare costs 1 2 . CADx-assisted optical diagnosis for real-time prediction of polyp histology might enable two key optical diagnosis-based management strategies: 1) “resect and discard,” where diminutive polyps deemed neoplastic are excised and discarded without histopathological assessment; and 2) “diagnose and leave,” where hyperplastic lesions in the rectosigmoid are left in situ following optical diagnosis 3 .

Several large studies have shown adequate diagnostic performance for CADx-assisted and CADx-unassisted optical diagnosis 4 5 6 . A recent study demonstrated the pragmatic implementation of resect and discard and diagnose and leave strategies using CADx-assisted optical diagnosis 7 . However, when implementing such optical diagnosis-based strategies, an independent video- or image-based audit of diagnostic performance becomes essential. European Society of Gastrointestinal Endoscopy guidelines recommend audit of polyps undergoing optical diagnosis 8 . However, methodology for conducting such audits and associated diagnostic accuracy compared with pathology remain unclear.

To address this knowledge gap, we evaluated audit performance in a prospective video-based study using video and image material from a large prospective study with systematic CADx-assisted optical diagnosis and video recording of all procedures. The study aim was to provide information on performance of diagnostic audit and offer a framework for how audit of CADx-assisted optical diagnosis should be performed.

## Patients and methods

This study is reported according to the Standards for Reporting of Diagnostic Accuracy Studies (STARD) checklist 9 . All authors had access to the study data and reviewed and approved the final manuscript.

### Study design and participants

This study was a prospective study using data from a previous prospective trial evaluating CADx-assisted optical polyp diagnoses 10 . Consecutive patients aged 45 to 80 years were enrolled at a single center (Montreal University Hospital Center) and underwent elective colonoscopy with CADx-assisted optical diagnosis. Consent for study participation and secondary use of data was obtained from all patients. Secondary data use and the audit evaluation subproject were preconceived in the original protocol and approved by the Institutional Review Board (CER 21.305; NCT05236790). Patient inclusion and exclusion criteria have been reported previously 10 .

### Study procedures

High-definition endoscopes (ELUXEO 7000 System, EC-760S-A/M and EC-760S-A/L colonoscopes; Fujifilm, Tokyo, Japan) were used for colonoscopies. The CAD-EYE software (EW10-EC02; Fujifilm) was used for imaging of all diminutive colorectal polyps (≤ 5 mm). Four board-certified gastroenterologists or one trainee performed all colonoscopies. The linked-color and blue-light imaging functions were activated to enhance visualization of polyp microscopic features. Polyp size was measured optically by the endoscopist or using the CAD-EYE integrated virtual eye scale (Scale-eye). Polyps were assessed using CADx-assisted optical diagnosis and full-length video recordings of the procedures were retained. All polyps were resected and sent to pathology for review by a single board-certified pathologist who specialized in gastrointestinal diseases. Polyps were cut into at least three levels and classified according to the World Health Organization classification system 11 .

In the current study, image and video sequences of each diminutive polyp were extracted from the colonoscopy recordings by research assistants (F.H., T.I.D., D.H.D.K, B.A., N.H., S.F.). All retrieved diminutive polyps from the index study were used for inclusion in this secondary analysis. The materials were incorporated into an online questionnaire using LimeSurvey (Hamburg, Germany), with each page exhibiting a different polyp. The survey was used for audit by three independent expert endoscopists (D.K.R., H.P., C.S.). Each expert had more than 10 years of endoscopy experience and participated in at least 10 prior optical diagnosis and CADx studies. Research assistants (F.H., T.I.D., D.H.D.K.) helped the experts navigate the survey. Experts were provided with images and short video sequences showing each polyp along with information on anatomical location and polyp size but were blinded to each other's diagnosis, CADx optical diagnosis, and pathology results. Experts classified each polyp as adenoma, hyperplastic, sessile serrated lesion (SSL), normal/mucosal prolapse, inflammatory, or unclear. They rated their confidence in their diagnosis as high or low. The experts also indicated whether overall quality of the image and video for each polyp was adequate or inadequate for audit. Quality documentation was determined by the “3 C” endoscopy photo quality checklist proposed by Ahmad et al. (clean mucosal surface, complete view, correct focal distance), as well as suitability of image modalities (white-light imaging, linked-color imaging, blue-light imaging) for polyp visualization 4 .

### Data collection and outcomes

Collected data included patient age, sex, American Society of Anesthesiologists class, Boston Bowel Preparation Scale score, and colonoscopy indication; polyp location, size, and histology; experts’ optical diagnoses, levels of confidence, and ratings of image and video quality. The primary outcome of the study was sensitivity of a three-expert review with high confidence, using histopathology assessment as the reference diagnosis. We hypothesized that sensitivity of histology prediction by experts would be superior to the 80% threshold defined by the ESGE Simple Optical Diagnosis Accuracy competence standards 12 . Secondary exploratory outcomes were: 1) to determine the optimal audit strategy for CADx-assisted optical diagnosis; and 2) expert interobserver agreement.

Audit strategy choices were:

- Strategy A - Review by three experts with concordant, high-confidence diagnoses;
- Strategy B - review by the first two experts (D.K.R., H.P.) with concordant, high-confidence diagnoses, with a referee (C.S.) in cases of disagreement;
- Strategy C -Two-expert audit, yielding three possible pairs of experts, because three experts participated in the study;
- Strategy D - Audit performed by each expert individually.

All strategies were evaluated and compared with each other using performance data (accuracy, sensitivity, specificity) and polyp sample sizes. Accuracy was defined as the number of polyps correctly diagnosed by the experts, all types combined, divided by the total number of polyps. Sensitivity was defined as the proportion of adenomas correctly diagnosed, and specificity was defined as the proportion of non-adenomatous polyps correctly identified as such.

### Sample size and statistical analysis

We assumed 90% sensitivity of expert audit when performed by three experts with concordant, high-confidence diagnoses. Therefore, to conduct a two-sided chi-squared test with a significance level of 5% and power of 80% to test whether the experts’ sensitivity was greater than 80%, it was necessary to include a minimum of 98 polyps with adenoma histology and concordant, high-confidence diagnosis by all experts.

Descriptive statistics are presented as numbers and frequencies for categorical variables, and as means with standard deviation (SD) for continuous variables. Diagnostic performance metrics were calculated for different combinations of experts with high-confidence diagnoses (i.e. for all three experts agreeing on the diagnosis (Strategy A), for two experts with a referee in cases of disagreement (Strategy B), for two experts agreeing on the diagnosis (Strategy C), and for each expert individually (Strategy D). For all metrics (accuracy, sensitivity, specificity), generalized estimating equations assuming exchangeable intra-patient correlation between the polyps led to an estimated correlation close to zero; hence, independence was assumed. Estimated overall accuracy, adenoma sensitivity and specificity are presented with 95% confidence intervals (CIs), and two-tailed chi-squared tests with a 5% significance level were considered significant for all analyses. Light’s kappa statistics were used to determine interobserver agreements. Analyses were performed using R 4.4.2 (R Core Team, 2024; R Foundation for Statistical Computing, Vienna, Austria).

## Results

### Patient and polyp characteristics

**Fig. 1:**
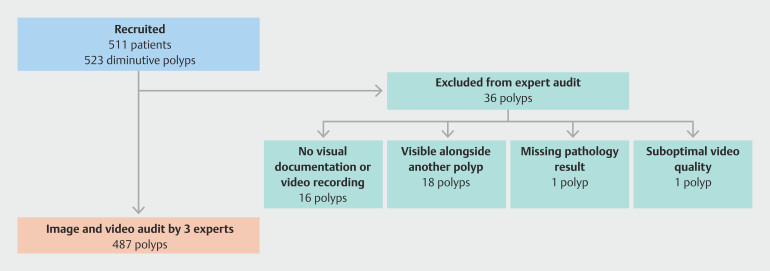
Study flow chart.

Among 510 patients, 523 diminutive (≤ 5 mm) colorectal polyps were identified, of which 36 polyps were excluded from the audit (18 because they were visible alongside another polyp, 16 because video recordings were missing, 1 because pathology results were not available, and 1 because of suboptimal video quality) ( Fig. 1 ). Finally, 487 polyps were independently reviewed by each of the three expert endoscopists, of which 266 (54.6%) were histologically diagnosed as adenomas, 130 (26.7%) as hyperplastic polyps, 16 (3.3%) as SSLs, and 63 (12.9%) as normal mucosa ( Table 1 ). Of the 266 adenomas, the three-expert audit reached concordant, high-confidence diagnosis for 160 polyps (60.2%). Two experts reported using images more frequently than videos for polyp diagnosis throughout the review, and one reported predominant use of videos. Pathology reported high-grade dysplasia in two of 487 reviewed polyps (0.4%), which went undetected by the experts.

**Table:** **Table 1** Patient and polyp characteristics.

Characteristics	
Patients, n	510
Age, mean (SD), years	64.0 (8.6)
Female, n (%)	254 (49.8)
Colonoscopy indication, n (%)	
Screening	72 (14.1)
Surveillance	274 (53.7)
Diagnostic	142 (27.8)
Polypectomy	16 (3.1)
Polyps included in expert audit, n	487
Size, mm, n (%)	
1–3	333 (68.4)
3–5	154 (31.6)
Polyp location, n (%)	
Cecum	50 (10.3)
Ascending	110 (22.6)
Hepatic flexure	2 (0.4)
Transverse	110 (22.6)
Descending	81(16.6)
Sigmoid	98 (20.1)
Rectum	36 (7.4)
Histology, n (%)	
Adenoma	266 (54.6)
Hyperplastic polyp	130 (26.7)
SSL	16 (3.3)
Normal mucosa	63 (12.9)
Inflammatory polyp	8 (1.6)
Other	4 (0.8)
HGD, n (%)	2 (0.4)
HGD, high-grade dysplasia; SSL, sessile serrated polyp.	

### Three-expert review sensitivity

**Fig. 2:**
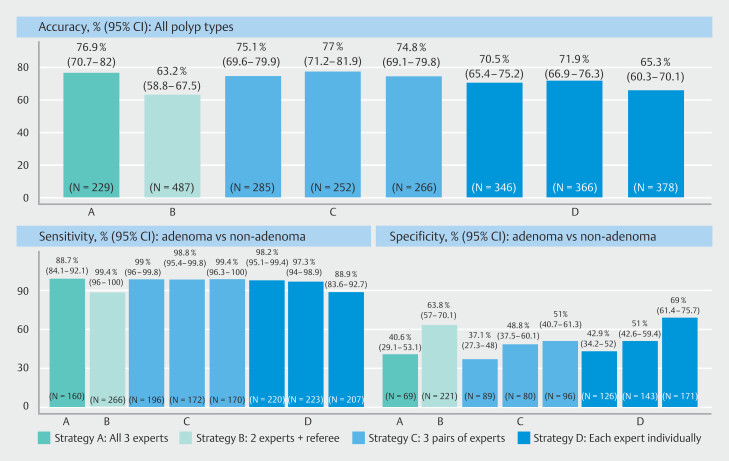
Diagnostic characteristics of each audit strategy (%, 95% confidence interval [CI]). For each performance metric and audit strategy, the number of polyps included in the denominator is indicated at the bottom of the panels.

Audit of adenomas by three experts with concordant, high-confidence diagnosis (Strategy A) yielded 99.4% sensitivity (159/160, 95%CI 96.0–100) ( Fig. 2 ). This sensitivity was found to be significantly higher than the 80% threshold defined by the ESGE guidelines ( *P* < 0.001).

### Choice of audit strategy

Overall expert accuracy was 76.9% (176/229, 95% CI 70.7–82.0) in Strategy A, whereas it was 63.2% (308/487, 95% CI 58.8–67.5) in a review by two experts and a referee (Strategy B) ( Fig. 2 ). The three possible pairs of experts in Strategy C had similar accuracies (75.1% [95% CI 69.6–79.9], 77% [95% CI 71.2–81.9], and 74.8% [95% CI 69.1–79.8]). In Strategy D, each expert individually achieved different levels of accuracy (70.5% [95% CI 65.4–75.2], 71.9% [95% CI 66.9–76.3], and 65.3% [95% CI 60.3–70.1]).

Sensitivity was 88.7% (236/266, 95%CI 84.1–92.1) in Strategy B, whereas sensitivities in Strategy C were 99.0% (194/196, 95%CI 96.0–99.8), 98.8% (170/172, 95%CI 95.4–99.8), and 99.4% (169/170, 95%CI 96.3–100). Strategies A and C thus yielded similar sensitivities with overlapping 95%CIs ( Fig. 2 ). Different sensitivities were obtained in Strategy D for each expert (98.2% [95% CI 95.1–99.4], 97.3% [95% CI 94.0–98.9], and 88.9% [95% CI 83.6–92.7]).

Specificity was 40.6% (95%CI 29.1–53.1) in Strategy A and was higher in Strategy B (63.8% [95% CI 57.0–70.1]). Specificities in Strategy C varied depending on the pair of experts (37.1% [95%CI 27.3–48.0], 48.8% [95%CI 37.5–60.1], and 51% [95%CI 40.7–61.3]). Specificities also varied significantly in Strategy D (42.9% [95% CI 34.2–52], 51% [95% CI 42.6–59.4], 69% [61.4–75.7]).

For each strategy, the number of experts influenced the number of polyps with concordant, high-confidence diagnoses that were included in performance assessments. Among all polyp types, Strategy B allowed inclusion of all polyps (N = 487), compared with 229 in Strategy A; 285, 252, and 266 in Strategy C; and 346, 366, and 378 in Strategy D. When calculating sensitivities and therefore only considering all adenomas (N = 266), Strategy A allowed inclusion of 160 polyps, versus 196, 172, and 170 polyps in Strategy C; and 220, 223, and 207 in Strategy D. In Strategy B, all 266 adenomas were included.

### Interobserver agreement

**Fig. 3:**
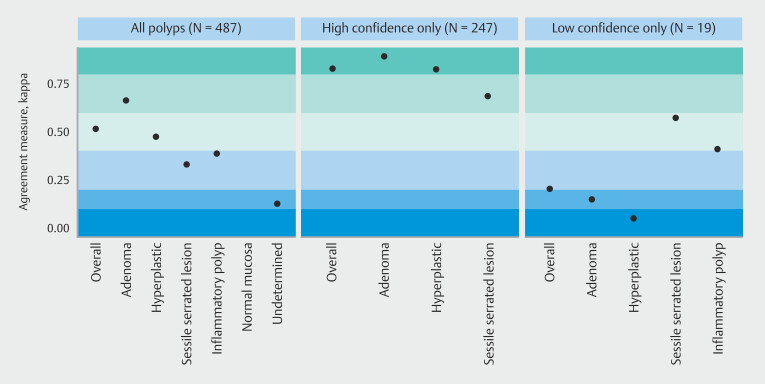
Expert interobserver agreement according to polyp histology and confidence level. Dark blue: no agreement; dark green: near perfect agreement.

Overall agreement between the three experts was moderate for all polyps, with a kappa value of 0.52, and substantial for adenomas (kappa 0.66) ( Fig. 3 ). Interobserver agreement for high-confidence diagnoses was very high, with a kappa value of 0.83 overall. More specifically, consensus was the highest for high-confidence diagnoses of adenomas (kappa 0.89), whereas it was lower for hyperplastic polyps (kappa 0.82) and even lower for SSLs (kappa 0.69).

## Discussion

In this prospective study, we found that sensitivity of expert review was very high for ≤ 5-mm polyps unanimously diagnosed as high-confidence adenomas. Regardless of the number of auditors, expert performance in diagnosing adenomas was found to be likely sufficient for audit of CADx-assisted optical diagnosis. Furthermore, our findings suggest that the ideal audit strategy is an initial high-confidence review by two blinded experts, with a third expert available for arbitration in cases of disagreement. Although this approach yielded lower accuracy and sensitivity compared with other strategies (three-expert audit, two-expert audits, single-expert audit), Strategy B allowed all polyps to be included, making it a more practical approach for auditing CADx. Moreover, specificity of Strategy B was higher than that of Strategies A and C, and was less affected by individual expert performances such as in Strategy D. Nevertheless, suboptimal specificity across all audit strategies highlights the tendency of endoscopists to over-diagnose adenomas, which has also been reported previously 13 . A significant proportion of pathology-based hyperplastic polyps or normal mucosa have likely been diagnosed as high-confidence adenomas by the experts. This phenomenon could be due to endoscopists’ fear of misdiagnosing adenomas as another benign lesion because of patient safety concerns. However, inaccurate histological diagnosis of high-confidence adenomas as normal mucosa due to errors in polyp retrieval and sectioning is an alternative hypothesis for the low audit specificity 4 14 . A study reported that among ≤ 3-mm colorectal lesions that were all endoscopically diagnosed as high-confidence adenomas by expert endoscopists, pathology reported up to 15% as normal mucosa 15 .

Although interobserver agreement was moderate overall, very high inter-expert agreement was found for high-confidence adenoma diagnoses. Our results underscore the necessity of stating a confidence level when performing optical diagnosis. Good image and video quality for documentation is essential for high-confidence diagnoses to be made. For all experts, high-confidence diagnoses were found to be significantly correlated with good quality polyp presentation ( *P* < 0.001) ( **Supplementary Table 1** ). Conversely, poor image and video quality and low confidence level could become a barrier for implementing expert audit. Stability of scope position during polyp visualization and adequate colonoscope set-up and training of endoscopists, therefore, are crucial to enhance picture quality. Moreover, artificial intelligence (AI) could eventually be used to provide real-time feedback to the endoscopist about quality of polyp visualization 16 . Further assessment of expert inter-observer agreement for picture quality documentation is required in future studies assessing CADx.

Overall expert agreement was lower than that reported in previous studies evaluating performance of experienced endoscopists in differentiating neoplastic from hyperplastic lesions, with kappa scores ranging from 0.67 to 0.90 17 18 . Inclusion of SSLs, normal mucosa, and inflammatory polyps in the expert review likely plays a role in this decrease in overall agreement; however, when foregoing histopathology within CADx implementation studies this seems to be important. For instance, difficulties frequently arise during endoscopic classification of hyperplastic polyps and SSLs owing to overlapping features between these polyp types, and this could lead to a lack of concordance within studies 1 19 . Despite recent development of criteria to help endoscopists optically diagnose SSLs, diagnostic accuracy remained low even after training 20 . Poor-to-moderate interobserver agreement between pathologists for differentiating these lesions has also been documented, with one study reporting a kappa value of 0.44 for SSL diagnosis 21 . Moreover, studies have shown moderate-to-high agreement among pathologists for differentiation of neoplastic versus hyperplastic polyps (kappa 0.67–0.89), which is not ideal considering its use as a gold standard diagnosis 22 23 24 . Therefore, our study suggests that a similar performance between expert review and pathology can be expected for diminutive polyp diagnosis even when including SSLs. Thresholds for interobserver agreement and diagnostic accuracy, sensitivity, and specificity should be further defined to ensure sufficient quality for audit of both optical diagnosis and CADx-assisted optical diagnosis.

Because several efforts are currently being made to implement optical diagnosis with the “resect and discard” strategy in real-world settings, an efficient and effective audit strategy becomes essential 7 . For instance, implementation has already begun within the Bowel Cancer Screening Programme in England, and cost and time savings have already been made 25 . To our knowledge, this is the first study to evaluate and support the feasibility of expert audit as a diagnostic reference for assessing CADx performance while also providing an optimal strategy for audit implementation. When adenoma diagnoses are concordant and performed with high confidence, audit by two experts and a third in case of disagreement could be used to measure diagnostic performance of optical diagnosis and CADx-assisted optical diagnosis in upcoming true implementation studies. Further research is needed to generalize the results to multiple experts, because only three were included in this study. Factors that influence the level of confidence in optical diagnosis during audit, namely image and video quality, also should be further analyzed. Moreover, optical review by expert endoscopists could avoid inaccurate histological diagnoses that occur frequently in pathology through errors in polyp retrieval, sectioning, and misdiagnoses as normal mucosa 4 . Errors in histopathology assessments could hinder training and testing of CADx programs by causing difficulties in supplying the “truth” to CADx algorithms 13 . It should be noted that evaluation of expert accuracy in this study was done by using histopathology assessment as the gold standard and was not adjusted for potentially inaccurate pathology results, namely among normal mucosa diagnoses. The calculated performance metrics of expert endoscopists could therefore be underestimated.

The primary strength of this study is inclusion of a large number of polyps for evaluation in a prospective audit. However, the study has several limitations. First, only three experts completed the audit, which limits generalizability of the results. Interobserver variability between individual expert accuracies could potentially affect validity of an expert audit of colorectal polyps. However, our study found that Strategy B, involving two experts completing the audit with a third expert available for arbitration if needed, standardized and increased specificity of the audit so that overall performance metrics remained satisfactory. Studies that compare accuracies of a larger number of experienced endoscopists from different countries are required to evaluate expert interobserver variability in polyp optical diagnosis. Second, intra-observer agreement for each expert was not assessed in this study. Diagnostic performance of each expert could potentially vary and affect overall performance of the expert audit if the review was to be later performed with the same set of polyps, warranting further evaluation. Previous studies have shown moderate-to-high intra-observer agreement for experts predicting polyp histology with a kappa score of 0.61–0.87, which was significantly lower than the perfect intra-observer agreement achieved by CADx 18 26 . Nevertheless, intra-observer agreement of pathologists for polyp histology has not been well documented in the literature 27 . In a study assessing intra-observer variability among experienced pathologists for colorectal adenomas, a kappa score of 0.41–0.90 was reported for determining histological subtype (tubular, tubulovillous, villous) and grade of dysplasia 28 . Third, the audit structure proposed in this study may be difficult to implement in routine clinical practice especially within large-scale audits, because using experts for manual polyp auditing is impractical and resource intensive. A cost-based analysis comparing expert review to traditional pathology should be performed in future studies to assess sustainability of an audit by expert endoscopists. Furthermore, a solution to impracticability of expert audit would be to train an independent AI model using still images of polyps. An AI-driven audit system could be used as the reference standard to assess accuracy of pathology, expert optical diagnosis, and current performance of CADx-assisted optical diagnosis. Similar to an audit by experts, an AI tool would help avoid inaccuracies that occur in pathology diagnoses due to errors in specimen retrieval and sectioning. Future studies should evaluate the feasibility of AI as an auditing tool to support the practical rollout of optical diagnosis with the “resect and discard” strategy.

## Conclusions

In summary, the findings from this study showed high sensitivity of expert review for diagnosing high-confidence adenomas that would be sufficient for assessing CADx-assisted optical diagnosis within clinical implementation studies. A review by two independent, blinded experts, with a third expert available for arbitration if needed, should be considered because it provided adequate diagnostic performance while including all polyps for audit, unlike the single-, two- and three-expert review with concordant, high-confidence diagnoses. Despite only moderate concordance between the experts overall, interobserver agreement was very high for high-confidence diagnoses, especially for adenomas.
